# Photodynamic Inactivation of SARS-CoV-2 Infectivity and Antiviral Treatment Effects In Vitro

**DOI:** 10.3390/v14061301

**Published:** 2022-06-14

**Authors:** Svitlana Ziganshyna, Grit Szczepankiewicz, Mathias Kuehnert, Agnes Schulze, Uwe Gerd Liebert, Corinna Pietsch, Volker Eulenburg, Robert Werdehausen

**Affiliations:** 1 Department of Anaesthesiology and Intensive Care, Medical Faculty, University of Leipzig, 04103 Leipzig, Germany; svitlana.ziganshyna@medizin.uni-leipzig.de (S.Z.); volker.eulenburg@medizin.uni-leipzig.de (V.E.); 2 Medical Faculty, Institute of Medical Microbiology and Virology, University of Leipzig, 04103 Leipzig, Germany; grit.szczepankiewicz@medizin.uni-leipzig.de (G.S.); liebert@medizin.uni-leipzig.de (U.G.L.); corinna.pietsch@medizin.uni-leipzig.de (C.P.); 3 Leibniz Institute of Surface Engineering (IOM), 04318 Leipzig, Germany; mathias.kuehnert@iom-leipzig.de (M.K.); agnes.schulze@iom-leipzig.de (A.S.)

**Keywords:** SARS, SARS-CoV-2, coronavirus, COVID-19, THPTS, photodynamic inactivation, photodynamic therapy, photosensitizer, near-infrared light

## Abstract

Despite available vaccines, antibodies and antiviral agents, the severe acute respiratory syndrome coronavirus-2 (SARS-CoV-2) pandemic still continues to cause severe disease and death. Current treatment options are limited, and emerging new mutations are a challenge. Thus, novel treatments and measures for prevention of viral infections are urgently required. Photodynamic inactivation (PDI) is a potential treatment for infections by a broad variety of critical pathogens, including viruses. We explored the infectiousness of clinical SARS-CoV-2 isolates in Vero cell cultures after PDI-treatment, using the photosensitizer Tetrahydroporphyrin-tetratosylate (THPTS) and near-infrared light. Replication of viral RNA (qPCR), viral cytopathic effects (microscopy) and mitochondrial activity were assessed. PDI of virus suspension with 1 µM THPTS before infection resulted in a reduction of detectable viral RNA by 3 log levels at day 3 and 6 after infection to similar levels as in previously heat-inactivated virions (<99.9%; *p* < 0.05). Mitochondrial activity, which was significantly reduced by viral infection, was markedly increased by PDI to levels similar to uninfected cell cultures. When applying THPTS-based PDI after infection, a single treatment had a virus load-reducing effect only at a higher concentration (3 µM) and reduced cell viability in terms of PDI-induced toxicity. Repeated PDI with 0.3 µM THPTS every 4 h for 3 d after infection reduced the viral load by more than 99.9% (*p* < 0.05), while cell viability was maintained. Our data demonstrate that THPTS-based antiviral PDI might constitute a promising approach for inactivation of SARS-CoV-2. Further testing will demonstrate if THPTS is also suitable to reduce the viral load in vivo.

## 1. Introduction

The still ongoing severe acute respiratory syndrome coronavirus type 2 (SARS-CoV-2) pandemic has so far impacted all domains of human life, especially healthcare, with well over 500 million confirmed infections and approaching 6.3 million infection-associated deaths globally reported to the World Health Organization (WHO) [1]. 

For SARS-CoV-2, aerosol and fomite transmission of infections is likely, since the virus can remain infectious in aerosols for hours and on surfaces up to days [2], and it is found even in wastewater [3]. Although vaccinations and antiviral treatment options like neutralizing antibodies have been developed and put into use, new mutational SARS-CoV-2 variants, such as the current omicron variants, as well as future viral threats, may not be susceptible to current treatment strategies. Therefore, new methods for the medical treatment of patients with COVID-19, as well as for prevention of SARS-CoV-2 infections and the protection of medical staff, are urgently required.

Photodynamic inactivation (PDI) is an emerging medical treatment and has the potential for treating critical infections caused by a broad variety of pathogens, including multi-resistant bacteria [4]. Recent reports also demonstrate the antiviral potential of PDI [5,6,7,8,9,10,11,12]. Antiviral PDI may therefore be a novel method for the treatment of contaminations and infections with SARS-CoV-2 and may also be helpful in preventing transmission of viral infections in blood component transfusions [13]. Moreover, photodynamic coatings have been demonstrated to be effective in reducing the pathogen-burden of patient-near surfaces and, therefore, the risk for onward pathogen transmission [14]. 

Here, PDI utilizes an entirely different mode of action than antiviral drugs, making PDI potentially effective even for pathogens that cannot be cured pharmacologically to date [15]. PDI requires three components: A photosensitizer (PS), molecular oxygen and light. While these components are individually non-toxic, together they can initiate a photochemical reaction to generate highly reactive oxygen species, such as singlet oxygen [16], which is very short-lived (1 ns) and therefore affects a very specific area (up to 0.1 μm). If generated at high concentrations within a tumor cell, this can lead to apoptosis, while on contaminated surfaces and infected tissue it can lead to inhibition of viral or bacterial infectivity, even at much lower concentrations.

Many PSs for PDT are based on porphyrinoids. Here, the vast spectrum of possible chemical modifications allows optimization and tailoring PS for specific applications, including improved water solubility, cellular penetration and application-optimized absorption spectra. Previous investigations have highlighted the effective inactivation of enveloped viruses, such as HIV [17] and influenza virus [18], by PDI based on porphyrin derivates. For this investigation, we focus on the porphyrin-based PS tetrahydroporphyrin-tetratosylate (THPTS), which offers favorable features like water solubility and safety. THPTS is best activated by near-infrared light (760 nm), which shows good tissue penetration (~1–2 cm) [19], and it can also be activated by bright white light, like sunlight. It would therefore be suitable for a broad spectrum of applications, for example, photodynamic coatings of frequently-touched surfaces even in low-tech/outside environments or for vulnerable surfaces that cannot be disinfected by usual methods. Additionally, direct antiviral therapy for acute respiratory syndromes, e.g., in COVID-19 patients may also be feasible, since the successful treatment of bronchial cancer by endoscopic illumination [20] has been described already, using porfimer sodium as a PS [21], which is approved for clinical application in Canada, Japan and USA. Another promising candidate is padeliporfin [22], which has been approved by the EU for the treatment of prostate cancer since April 2018. Both clinically approved PS can be applied topically as well as intravenously. 

To our knowledge, the usefulness of THPTS-based PDI in SARS-CoV-2 contaminations and infections has not been investigated. Recently, however, the first reports of successful PDI treatments against SARS-CoV-2 or surrogates in vitro have emerged [23,24,25,26]. Here, we report the effects of THPTS-based PDI on SARS-CoV-2 infectivity as well as treatment effects in infected cell cultures.

## 2. Materials and Methods

The photosensitizer 3,3′,3″,3‴-(7,8,17,18-tetrahydro-21H,23H-porphyrine-5,10,15,20-tetrayl)tetrakis [1-methyl-pyridinium]tetratosylate, trivial name of tetrahydroporphyrintetratosylate (THPTS) (C_72_H_70_N_8_O_12_S_4_, molecular weight 1367.66 Da, purity ≥ 95%), was purchased from TetraPDT Inc. (Rackwitz, Germany) and used for all photodynamic inactivation experiments. A detailed characterization was published previously [27]. A stock solution (10 mM) of THPTS in PBS was prepared and stored in the dark at 4 °C for up to 30 days prior to use.

### 2.1. SARS-CoV-2 Isolation and Propagation of Viral Suspensions

All virus cultures and assays were carried out in a biosafety level-3 laboratory. All virus infection experiments were performed in Vero E6 kidney epithelial cells from an African green monkey (CCLV-RIE 0929; Collection of Cell Lines in Veterinary Medicine) expressing the ACE-2 receptor, as the infectivity of SARS-CoV-2 strictly depends on ACE-2. Vero E6 cells were propagated in 75 cm^2^ cell culture flasks in a growth medium consisting of DMEM (Life Technologies Europe, Merelbeke, Belgium), supplemented with 5% fetal calf serum (FCS) and penicillin/streptomycin. The identity of the used SARS-CoV-2 isolate (SARS-CoV-2/human/Germany/LE-B00KXK2/2020 from a SARS-CoV-2 patient in Leipzig, Germany) was confirmed by sequencing. For virus propagation, 2 mL of stock virus was added to a confluent monolayer of Vero E6 cells and incubated at 37 °C in 5% CO_2_ for 1 h; 13 mL of DMEM supplemented with 5% FCS was then added. The cultures were incubated at 37 °C in 5% CO_2_, and the supernatant was harvested after 48 h when cytopathic effects (CPE) were observed with an inverted microscope. The supernatant was clarified by centrifugation at 200× *g* for 10 min at 20 °C and then divided into samples for PDI-treatment. The virus titer in the obtained culture supernatants was determined by using qPCR. 

### 2.2. Photodynamic Inactivation Treatment

A custom-made light-emitting diode (LED) array with the required wavelength of ʎ = 760 nm for THPTS excitation was used, powered by ahigh-current power supply (PowerPac HC; Bio-Rad; Hercules, CA, USA). The total light energy output of the LED array was adjusted with an optical multimeter (ILX Lightwave OMM-6810B) and an optical power measurement head (OMH-6720B). The setup was adjusted for a homogenously applied energy to the sample of 13 mW/cm^2^ at an LED array-to-sample distance of 8 cm [28].

For PDI experiments, the viral suspension was pipetted into sterile FACS tubes at a volume of 500 µL per sample. THPTS was added (5 µL), and after vortexing for 30 s and a total preincubation time of 4 min in the dark, the samples were illuminated. The final THPTS concentration was set to 0.1, 0.3 or 1 µM, as indicated in results. The illumination duration was set to 5 or 10 min (3.9 or 7.8 J/cm^2^) as indicated.

Additional samples were prepared as dark toxicity controls. These were performed with the same test setup, including preincubation with THPTS, but without illumination. Further controls were performed using a vehicle (PBS) instead of THPTS, followed by standard illumination treatment (light controls). All experimental conditions were performed in triplicates.

### 2.3. Viral RNA PCR

Nucleic acid extraction and PCR preparation for qualitative and quantitative PCRs were performed by means of the fully automated AltoStar AM16 (Altona Diagnostics, Hamburg, Germany) using the AltoStar^®^ Purification Kit 1.5, including AltoStar^®^ Internal Control 1.5 (Altona Diagnostics). Real-time PCR plates pipetted through the instrument were analyzed in a CFX96 cycler from Bio-Rad using the AltoStar^®^ SARS-CoV-2 RT PCR Kit 1.5 (Altona Diagnostics).

### 2.4. Infection Treatment

For infection experiments, 500 µL of a PDI-treated virus suspension was added to a confluent monolayer of Vero E6 cells and incubated at 37 °C in 5% CO_2_ for 1 h. Then, the viral suspension was removed and replaced by a fresh cell culture medium. The cultures were incubated at 37 °C in 5% CO_2_, and the supernatant was harvested 4 and 6 days after infection (4/6 dpi). Evaluation of the cytopathic effect in each well was analyzed with an inverted light microscope. At the day of harvest, the supernatants of the cells were collected and clarified by centrifugation at 200× *g* for 10 min at 20 °C. The virus titer in the obtained culture supernatants was determined by using qPCR. For heat-inactivation, the viral suspension was incubated at 56 °C for 15 min. 

### 2.5. Statistical Analysis

Data are expressed as the mean ± SD. Differences between groups were tested by a two-way ANOVA with Dunnet’s post hoc test. Calculations were performed using the Graph Pad Prism Software version 9.3.1 (GraphPad Software Inc., La Jolla, CA, USA). A *p* value of < 0.05 was considered indicative for significant differences.

## 3. Results

### 3.1. Effect of PDI on SARS-CoV-2 Infectivity

To investigate the effects of a THPTS-based PDI on the infectivity of SARS-CoV-2, we preincubated viral suspensions of SARS-CoV-2 with the photosensitizer THPTS at concentrations of 0.1, 0.3 and 1 µM or a vehicle for 4 min before exposition to near-infrared light (760 nm) for 10 min. Then, we performed an infection procedure with these pretreated viral suspensions in ACE2-expressing Vero E6 cell cultures.

**Figure 1:**
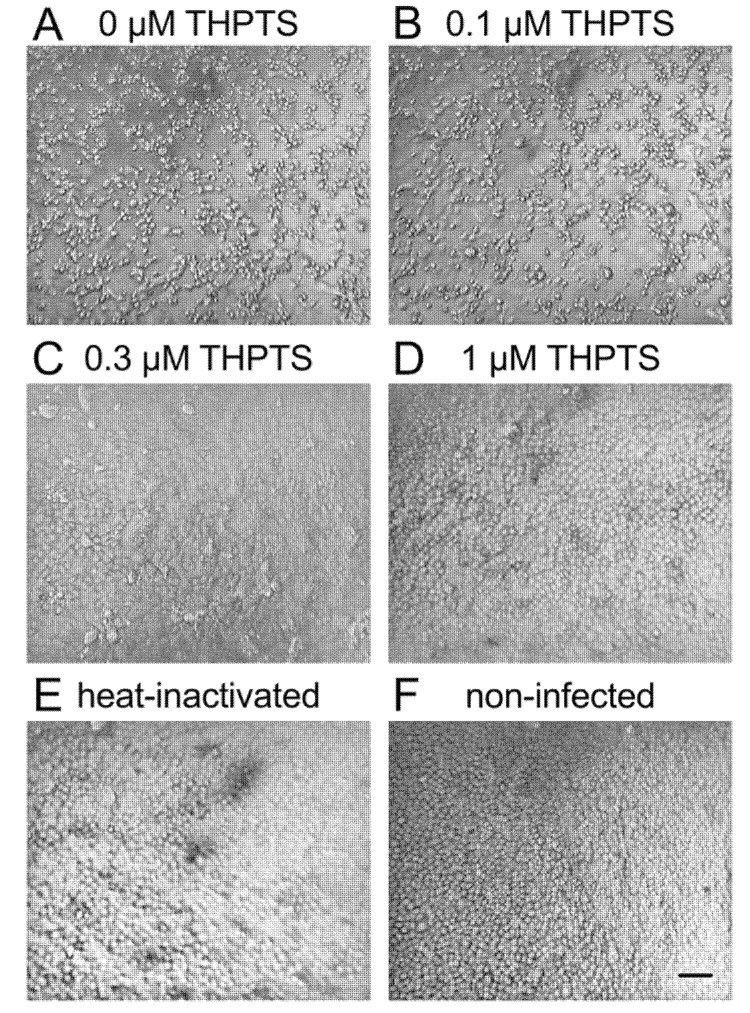
**Viral cytopathic effects after infection of Vero E6 cells with PDI-treated SARS-CoV-2.** Six days after infection, cell cultures showed clear signs of viral cytopathic effects (CPE) when photodynamic inactivation (PDI) of SARS-CoV-2 was performed for 10 min (light dose 7.8 J/cm^2^) without the photosensitizer THPTS prior to infection (**A**). With THPTS at indicated concentrations (**B**–**D**), the PDI of SARS-Cov-2 prevented subsequent infection and, therefore, CPE in a concentration-dependent manner. At 1 µM THPTS (**D**), cell cultures were free from signs of viral infection and very similar to those after infection with heat-inactivated SARS-CoV-2 (**E**) and those without infection (**F**). Scale bar indicates 100 µm.

In parallel processed cell cultures that were infected with untreated viral suspensions or with virus suspensions that were previously exposed to light but not treated with THPTS, evaluation by light microscopy six days after infection (dpi) revealed clear signs of viral cytopathic effects (Figure 1A and data not shown).

THPTS-based PDI led to a visible concentration-dependent reduction of viral cytopathic effects at 0.3 and 1 µM THPTS. At 1 µM THPTS, cell cultures appeared indistinguishable from mock-infected cell cultures (with heat-inactivated SARS-CoV-2) and those without infection (Figure 1B–F).

**Figure 2:**
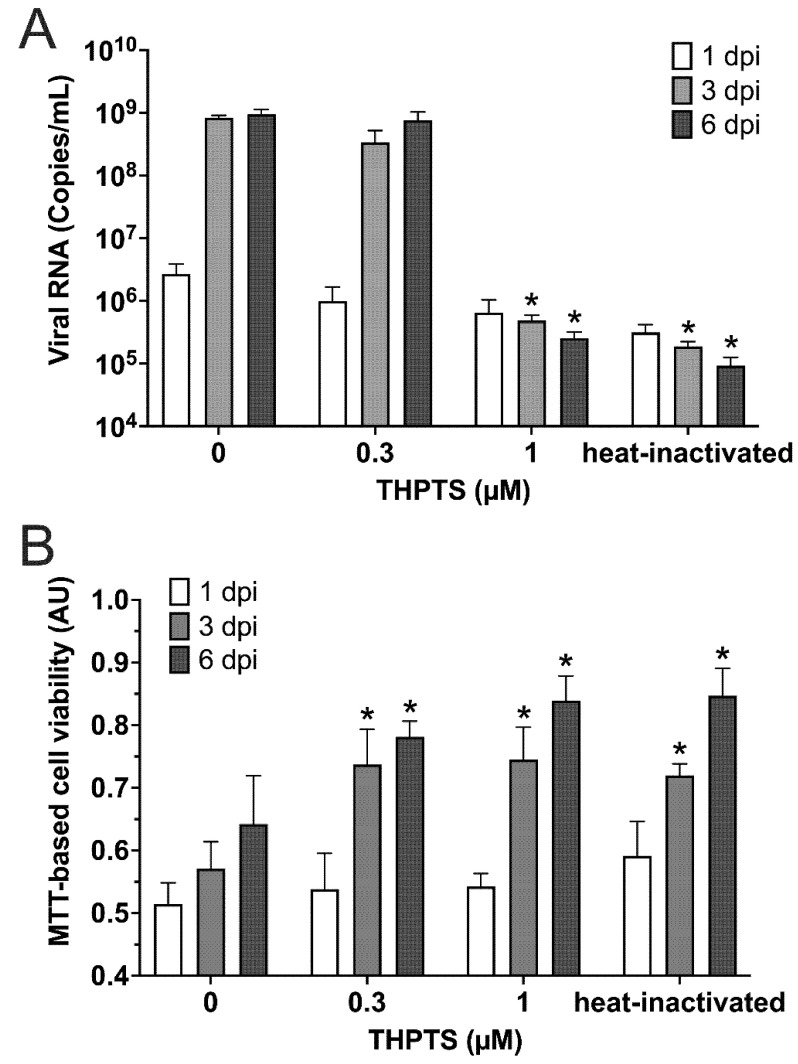
**Results from photodynamic inactivation treatment of SARS-CoV-2.** (**A**) Viral RNA was detected in supernatants after PDI-treatment for 10 min (light dose 7.8 J/cm^2^) at different photosensitizer (PS) concentrations or after heat-inactivation and subsequent infection (1/3/6 dpi; *n* = 4) of Vero cell cultures. (**B**) Cell viability was determined by an MTT-based assay for mitochondrial activity. Data are presented as mean and SD. * *p* < 0.05 when compared to respective samples treated with 0 µM THPTS.

When detecting the number of viral RNA copies at 3 dpi, it was drastically reduced by more than 99.90% (*p* < 0.05) when 1 µM THPS and 10 min of light application were combined for PDI (Figure 2A).

This result obtained here was similar to that of samples treated with heat-inactivated viral suspension used for infection. In both samples, viral RNA copy numbers were found to be reduced by 99.97% (*p* < 0.05; Figure 2A). When investigating the same cell cultures three days later (6 dpi), very similar results were obtained, indicating a sustained prevention of viral infectivity, consistent with a complete inactivation of SARS-CoV-2 virions in these samples. In contrast, controls that were treated with THPTS at concentrations of up to 100 µM and incubated up to 100 min in the dark (without irradiation activation) did not show a reduction of viral RNA copies (data not shown).

For evaluation of cell viability in the SARS-CoV-2-infected Vero cell cultures, we applied the MTT-assay for detection of mitochondrial activity. We observed that a single PDI treatment with at least 0.3 µM THPTS and 10 min illumination of virus suspensions prior to cell culture infection led to a significant increase of cell viability three and six days after infection (Figure 2B) when compared to samples infected with virus suspensions that were treated with illumination only (0 µM THPTS, *p* < 0.05). Taken together, these data demonstrate that PDI treatment with THPTS of SARS-CoV-2 virus suspensions resulted in an efficient inhibition of virus amplification and amelioration of viral CPE.

### 3.2. Effects of PDI on Cell Cultures Preinfected with SARS-CoV-2

To investigate if THPTS-based PDI can also reduce virus propagation and CPE at later stages, i.e., after initial infection, we performed PDI treatment in already SARS-CoV-2-infected cell cultures. Here, Vero E6 cell cultures were infected at low multiplicity of infection (MOI 0.005 and 0.01) in order to achieve an only marginal rate of initial infection. One hour after infection, PDI treatment was performed either once or recurring every 4 h for 3 days.

**Figure 3:**
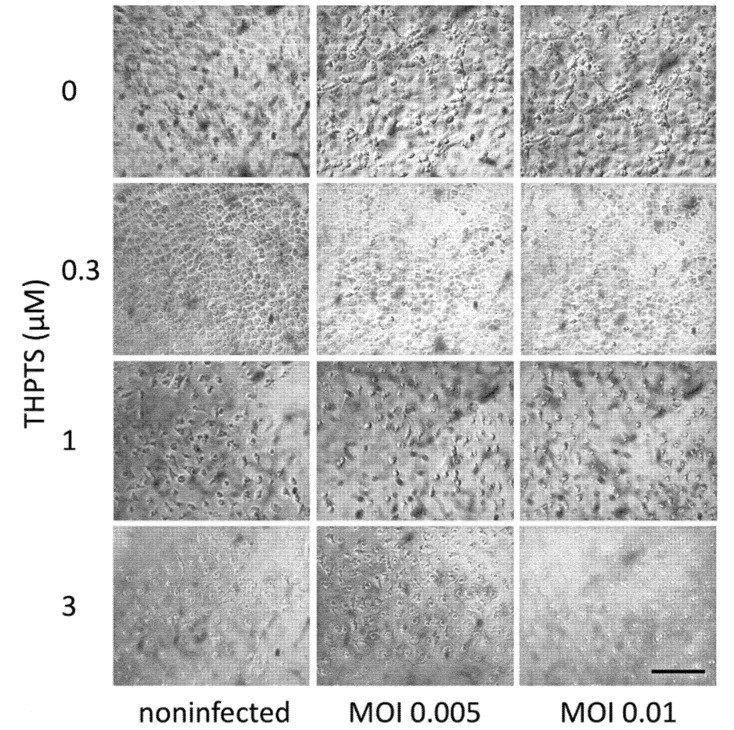
**Viral cytopathic effects in Vero E6 cells after infection with SARS-CoV-2 and subsequent (single) PDI treatment.** Three days after infection, cell cultures showed clear signs of viral cytopathic effects as a consequence of SARS-CoV-2 infection. When infection was followed by one single PDI for 10 min (light dose 7.8 J/cm^2^) with THPTS at indicated concentrations, viral cytopathic effects were mitigated in a concentration-dependent manner. At 1 µM THPTS, toxic effects of the PDI treatment were observed. Scale bar indicates 100 µm.

Vero E6 cell cultures infected with untreated virus suspension revealed typical signs of viral cytopathology 3 days after SARS-CoV-2-infection could be detected by light microscopy. Similar CPEs were observed in tissue cultures that received only light treatment once after infection (Figure 3). After single PDI with 0.3 µM THPTS, the viral CPEs were reduced, while higher concentrations of THPTS (1–3 µM) caused morphologic signs of toxic PDI effects.

**Figure 4:**
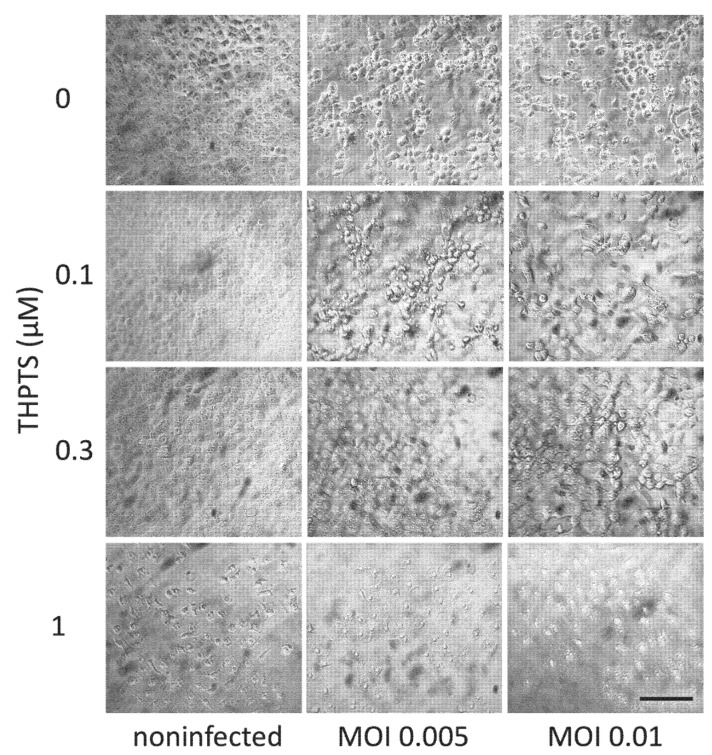
**Viral cytopathic effects in Vero E6 cells after infection with SARS-CoV-2 and subsequent repeated PDI treatment.** Three days after infection, cell cultures showed clear signs of viral cytopathic effects as a consequence of SARS-CoV-2 infection. When infection was followed by repeated PDI for 5 min every 4 h (light dose 3.9 J/cm^2^) with THPTS at indicated concentrations, viral cytopathic effects were mitigated in a concentration-dependent manner. At 1 µM THPTS, toxic effects of the PDI treatment were observed. Scale bar indicates 100 µm.

Repeated PDI with THPTS concentrations of 0.1 µM partially and 0.3 µM completely prevented CPE signs when compared to non-infected cell cultures, while PDI with 1 µM THPTS and above caused significant changes in cell morphology, most likely due to the cytotoxicity of the PDI treatment under these conditions (Figure 4).

**Figure 5:**
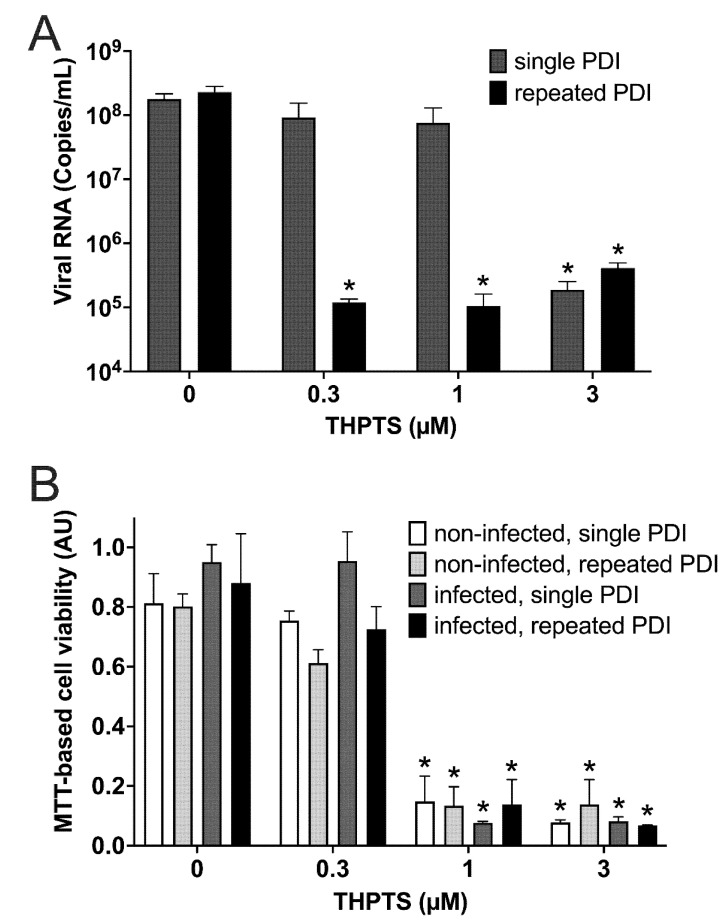
**Results from photodynamic inactivation treatment of Vero cell cultures after infection with SARS-CoV-2.** (**A**) Viral RNA was detected after SARS-CoV-2 infection of Vero cell cultures and subsequent single (5 min) or repeated PDI (5 min every 4 h for 3 d) at different photosensitizer (PS) concentrations. (**B**) Cell viability was determined by an MTT-based assay for mitochondrial activity. Data are presented as mean and SD. * *p* < 0.05 when compared to respective samples treated with 0 µM THPTS.

When analyzing viral replication at 3 dpi with viral PCR, we found a reduced number of viral copies after single PDI only in samples treated with 3 µM THPTS, while single PDI with lower concentrations of THTPS had no significant effect on viral replication in infected cell cultures (Figure 5A). However, after repeated PDI (every 4 h), we found a significant decrease of viral copy numbers already at lower concentrations of THPTS (0.3 µM and 1 µM). Consistent with our light microscopic data, we observed uncompromised mitochondrial activity after single and repeated PDI treatment with up to 0.3 µM of THPTS when compared to samples treated without THPTS (Figure 5B).

## 4. Discussion

To our knowledge, this is the first study to describe the effects of THPTS-based PDI on SARS-CoV-2. Our results indicate that a short PDI treatment based on the photosensitizer THPTS and near-infrared LED light for 10 min was sufficient to completely mitigate subsequent infection of Vero E6 cell cultures, without any undesired effects on cell viability in infected cultures.

When performing a PDI treatment in previously SARS-CoV-2-infected cell cultures, we found that a repeated PDI treatment with 0.3 µM was sufficient to inhibit viral replication and prevented signs of viral cytopathology, while higher THPTS concentrations (1 µM) caused cytotoxicity when repeatedly activated by light. Although these data suggest a rather small therapeutic window between the lowest effective and the highest non-toxic concentration, one should keep in mind that our experiment was optimized for PCR-based detection of SARS-CoV-2. Furthermore, the Vero E6 cells used in this study have been described to be especially sensitive to cytotoxic effects [29]. Clinical applications of PDI based on THPTS and similar compounds have indicated a much lower potential of toxic effects on host tissues. In a clinical application, shorter preincubation intervals, for example, would most likely help to prevent undesired effects on host cells by limiting uptake while not reducing antiviral efficacy.

If we compare required concentrations of THTPS and durations of PDI-activating illumination with those required for the inactivation of bacteria, especially multi-drug-resistant critical Gram-negative strains like *E. coli* and *Kleb. pneumoniae*, we achieved inhibition of SARS-CoV-2 infectivity with much lower concentrations (0.3–1 µM compared to 200–400 µM) and much shorter illumination times (5–10 min compared to more than 100 min) [27,30,31,32]. This may be caused by a higher susceptibility of virions to oxidative stress, since they lack systems that protect them from oxidative damage. Moreover, it was suggested that part of the antibacterial PDI effect is elicited by a photosensitizer that has been taken up by the bacteria and thus acts inside the bacterial cell to exert its antimicrobial action [33,34]. In contrast, a superficial mode-of-action is more likely in virions. Here, it is conceivable that an oxidative modification, e.g., of theSARS-CoV-2 spike protein, occurs comparably quickly and might therefore inhibit receptor recognition and the cell membrane fusion process [35]

Consistent with this idea, it was reported that the high susceptibility of viruses to PDI is not only caused by reduced structural integrity of viral RNA and virion membranes, but by loss of their surface glycoproteins, leading to non-infectious “bald” virions [18]. Since SARS-CoV-2 requires an envelope homotrimeric spike glycoprotein (S) to interact with the cellular receptor ACE-2 [36], it was proposed that selective impairment of surface proteins, like spike glycoprotein (S), by PDI can effectively inhibit infectivity of SARS-CoV-2 [24,25,37]. Additionally, it was suggested that effectivity of antiviral PDI may, in part, be explained by a light-induced photocleavage reaction of viral ssRNA [38]. However, this occurs by light irradiation with much shorter wavelengths (345–420 nm). At those THPTS concentrations that effectively inhibited SARS-CoV-2 infectivity by PDI in our study (0.3–1 µM), the integrity of viral RNA remained unchanged. Only at much higher concentrations (100 µM), a slight reduction of detectable viral RNA copies was observed in viral suspensions after THPTS-based PDI (data not shown).

Within the last year, PDI effects on SARS-CoV-2 have been reported by several groups using a variety of photosensitizers [9,23,24,26,39,40,41]. Most of these reports focus on the use of methylene blue or curcumin, requiring blue light for activation, which may pose significant risks and does not penetrate into deeper tissues. Often, surrogates (like Phage Phi6 [42] or HIV-based pseudotyped models [43], lentiviruses pseudotyped with the SARS-CoV-2 Spike [44], murine hepatitis virus [38], alphacoronavirus HCoV-229E or the betacoronavirus HCoV-OC43 [44]) were used instead of SARS-CoV-2 isolates.

One recent study reported the effects of the octacationic phthalocyanine derivative octakis(cholinyl)zinc phthalocyanine [25], which, similarly to the bacteriochlorin THPTS used in our study, is a nitrogen/carbon-based macrocyclic compound that is positively charged and activated by light in the far-red spectrum (692 nm). They reported on the infectivity of SARS-CoV-2 isolates and found comparable results. In addition, they suggested oxidative modification of the spike protein as the underlying mechanism. However, they only performed PDI treatment of viral suspensions prior to infection of cell cultures. As we demonstrated, repeated PDI can also be applied after infections in order to prevent spread of infections and subsequent viral cytopathic effects.

Our investigation has been performed purely in vitro, and therefore, we cannot confirm clinical effectivity and safety. Antiviral PDI is not yet an established method in the clinical setting and, as by now, is mostly limited to topical applications. Recent reports suggest, however, that antiviral PDI has been successfully used in the clinical setting, e.g., for oral decontamination [45] and treatment of orofacial manifestations [46] in patients suffering from COVID-19. In these reports, clinically approved agents, like methylene blue or curcumin, are used as photosensitizers, although their properties are far from optimal.

## 5. Conclusions

We conclude that an antiviral PDI based on the photosensitizer THPTS and near-infrared LED light could be utilized to prevent SARS-CoV-2 infectivity. Moreover, an optimized PDI can be used to treat SARS-CoV-2 infections in vitro. The data we have obtained were from in vitro experiments only. Thus, the logical next step is the verification of these findings and exploration of effectivity and safety of THPTS-based PDI treatment in vivo. Here, prophylactic PDI treatments of fluids and surfaces may prevent transduction of SARS-CoV-2 infections in animal infection models. In addition, repeated antiviral PDI based on THPTS may also be able to prevent systemic infections when applied shortly after a potential exposure or even reduce viral load in the respiratory tract in progressed infections and, thus, may result in a slowing of disease progression or attenuation of disease symptoms.

Effects of antiviral PDI are not limited to SARS-CoV-2, but they may also constitute a promising tool for the inactivation of other up-to-now unknown viruses and might therefore prove useful in future pandemics.

## Data Availability

Data are contained within the article.

## References

[1] World Health Organization (2022). WHO COVID-19 Dashboard. https://covid19.who.int.

[2] van Doremalen N., Bushmaker T., Morris D.H., Holbrook M.G., Gamble A., Williamson B.N., Tamin A., Harcourt J.L., Thornburg N.J., Gerber S.I. (2020). Aerosol and Surface Stability of SARS-CoV-2 as Compared with SARS-CoV-1. N. Engl. J. Med..

[3] Lodder W., de Roda Husman A.M. (2020). SARS-CoV-2 in Wastewater: Potential Health Risk, but Also Data Source. Lancet Gastroenterol. Hepatol..

[4] Abrahamse H., Hamblin M.R. (2016). New Photosensitizers for Photodynamic Therapy. Biochem. J..

[5] Wainwright M. (2021). Anti-Infective Dyes in the Time of COVID. Dyes Pigm..

[6] Trigo-Gutierrez J.K., Vega-Chacón Y., Soares A.B., Mima E.G. (2021). de O. Antimicrobial Activity of Curcumin in Nanoformulations: A Comprehensive Review. Int. J. Mol. Sci..

[7] Yu S., Sun G., Sui Y., Li H., Mai Y., Wang G., Zhang N., Bi Y., Gao G.F., Xu P. (2021). Potent Inhibition of Severe Acute Respiratory Syndrome Coronavirus 2 by Photosensitizers Compounds. Dyes Pigm..

[8] Fekrazad R., Asefi S., Pourhajibagher M., Vahdatinia F., Fekrazad S., Bahador A., Abrahamse H., Hamblin M.R. (2021). Photobiomodulation and Antiviral Photodynamic Therapy in COVID-19 Management. Adv. Exp. Med. Biol..

[9] Conrado P.C.V., Sakita K.M., Arita G.S., Galinari C.B., Gonçalves R.S., Lopes L.D.G., Lonardoni M.V.C., Teixeira J.J.V., Bonfim-Mendonça P.S., Kioshima E.S. (2021). A Systematic Review of Photodynamic Therapy as an Antiviral Treatment: Potential Guidance for Dealing with SARS-CoV-2. Photodiagn. Photodyn. Ther..

[10] Lebedeva N.S., Gubarev Y.A., Koifman M.O., Koifman O.I. (2020). The Application of Porphyrins and Their Analogues for Inactivation of Viruses. Molecules.

[11] Sabino C.P., Ball A.R., Baptista M.S., Dai T., Hamblin M.R., Ribeiro M.S., Santos A.L., Sellera F.P., Tegos G.P., Wainwright M. (2020). Light-Based Technologies for Management of COVID-19 Pandemic Crisis. J. Photochem. Photobiol. B.

[12] Almeida A., Faustino M.A.F., Neves M.G.P.M.S. (2020). Antimicrobial Photodynamic Therapy in the Control of COVID-19. Antibiotics.

[13] Wasiluk T., Rogowska A., Boczkowska-Radziwon B., Zebrowska A., Bolkun L., Piszcz J., Radziwon P. (2021). Maintaining Plasma Quality and Safety in the State of Ongoing Epidemic—The Role of Pathogen Reduction. Transfus. Apher. Sci..

[14] Eichner A., Holzmann T., Eckl D.B., Zeman F., Koller M., Huber M., Pemmerl S., Schneider-Brachert W., Bäumler W. (2020). Novel Photodynamic Coating Reduces the Bioburden on Near-Patient Surfaces Thereby Reducing the Risk for Onward Pathogen Transmission: A Field Study in Two Hospitals. J. Hosp. Infect..

[15] St Denis T.G., Dai T., Izikson L., Astrakas C., Anderson R.R., Hamblin M.R., Tegos G.P. (2011). All You Need Is Light: Antimicrobial Photoinactivation as an Evolving and Emerging Discovery Strategy against Infectious Disease. Virulence.

[16] Hamblin M.R. (2016). Antimicrobial Photodynamic Inactivation: A Bright New Technique to Kill Resistant Microbes. Curr. Opin. Microbiol..

[17] Sadraeian M., da Cruz E.F., Boyle R.W., Bahou C., Chudasama V., Janini L.M.R., Diaz R.S., Guimarães F.E.G. (2021). Photoinduced Photosensitizer–Antibody Conjugates Kill HIV Env-Expressing Cells, Also Inactivating HIV. ACS Omega.

[18] Korneev D., Kurskaya O., Sharshov K., Eastwood J., Strakhovskaya M. (2019). Ultrastructural Aspects of Photodynamic Inactivation of Highly Pathogenic Avian H5N8 Influenza Virus. Viruses.

[19] Schastak S., Jean B., Handzel R., Kostenich G., Hermann R., Sack U., Orenstein A., Wang Y.-S., Wiedemann P. (2005). Improved Pharmacokinetics, Biodistribution and Necrosis in Vivo Using a New near Infra-Red Photosensitizer: Tetrahydroporphyrin Tetratosylat. J. Photochem. Photobiol. B Biol..

[20] Mehta H.J., Biswas A., Fernandez-Bussy S., Pipkin M., Machuca T., Jantz M.A. (2019). Photodynamic Therapy for Bronchial Microscopic Residual Disease After Resection in Lung Cancer. J. Bronchol. Interv. Pulmonol..

[21] Moghissi K., Dixon K., Stringer M., Thorpe J.A.C. (2009). Photofrin PDT for Early Stage Oesophageal Cancer: Long Term Results in 40 Patients and Literature Review. Photodiagn. Photodyn. Ther..

[22] Azzouzi A.-R., Vincendeau S., Barret E., Cicco A., Kleinclauss F., van der Poel H.G., Stief C.G., Rassweiler J., Salomon G., Solsona E. (2017). Padeliporfin Vascular-Targeted Photodynamic Therapy versus Active Surveillance in Men with Low-Risk Prostate Cancer (CLIN1001 PCM301): An Open-Label, Phase 3, Randomised Controlled Trial. Lancet Oncol..

[23] Arentz J., von der Heide H.-J. (2022). Evaluation of Methylene Blue Based Photodynamic Inactivation (PDI) against Intracellular B-CoV and SARS-CoV2 Viruses under Different Light Sources in Vitro as a Basis for New Local Treatment Strategies in the Early Phase of a Covid19 Infection. Photodiagn. Photodyn. Ther..

[24] Lobo C.S., Rodrigues-Santos P., Pereira D., Núñez J., Trêpa J.C.D., Sousa D.L., Lourenço J.V., Coelho M.F., de Almeida L.P., da Cunha J.S. (2022). Photodynamic Disinfection of SARS-CoV-2 Clinical Samples Using a Methylene Blue Formulation. Photochem. Photobiol. Sci..

[25] Sharshov K., Solomatina M., Kurskaya O., Kovalenko I., Kholina E., Fedorov V., Meerovich G., Rubin A., Strakhovskaya M. (2021). The Photosensitizer Octakis(Cholinyl)Zinc Phthalocyanine with Ability to Bind to a Model Spike Protein Leads to a Loss of SARS-CoV-2 Infectivity In Vitro When Exposed to Far-Red LED. Viruses.

[26] Svyatchenko V.A., Nikonov S.D., Mayorov A.P., Gelfond M.L., Loktev V.B. (2021). Antiviral Photodynamic Therapy: Inactivation and Inhibition of SARS-CoV-2 in Vitro Using Methylene Blue and Radachlorin. Photodiagn. Photodyn. Ther..

[27] Ziganshyna S., Guttenberger A., Lippmann N., Schulz S., Bercker S., Kahnt A., Rüffer T., Voigt A., Gerlach K., Werdehausen R. (2020). Tetrahydroporphyrin-Tetratosylate (THPTS)-Based Photodynamic Inactivation of Critical Multidrug-Resistant Bacteria in Vitro. Int. J. Antimicrob. Agents.

[28] Habermann N., Wachs M., Schulz S., Werdehausen R., Schwarz U.T. (2019). Development and Characterization of Planar LED Arrays for Medical Applications. Jpn. J. Appl. Phys..

[29] Menezes C., Valério E., Dias E. (2013). The Kidney Vero-E6 Cell Line: A Suitable Model to Study the Toxicity of Microcystins.

[30] Schastak S., Ziganshyna S., Gitter B., Wiedemann P., Claudepierre T. (2010). Efficient Photodynamic Therapy against Gram-Positive and Gram-Negative Bacteria Using THPTS, a Cationic Photosensitizer Excited by Infrared Wavelength. PLoS ONE.

[31] Glass S., Kühnert M., Lippmann N., Zimmer J., Werdehausen R., Abel B., Eulenburg V., Schulze A. (2021). Photosensitizer-Loaded Hydrogels for Photodynamic Inactivation of Multirestistant Bacteria in Wounds. RSC Adv..

[32] Lehnig M., Glass S., Lippmann N., Ziganshyna S., Eulenburg V., Werdehausen R. (2022). Evaluation of a Luminometric Cell Counting System in Context of Antimicrobial Photodynamic Inactivation. Microorganisms.

[33] Pereira M.A., Faustino M.A.F., Tomé J.P.C., Neves M.G.P.M.S., Tomé A.C., Cavaleiro J.A.S., Cunha Â., Almeida A. (2014). Influence of External Bacterial Structures on the Efficiency of Photodynamic Inactivation by a Cationic Porphyrin. Photochem. Photobiol. Sci..

[34] Hurst A.N., Scarbrough B., Saleh R., Hovey J., Ari F., Goyal S., Chi R.J., Troutman J.M., Vivero-Escoto J.L. (2019). Influence of Cationic Meso-Substituted Porphyrins on the Antimicrobial Photodynamic Efficacy and Cell Membrane Interaction in Escherichia Coli. Int. J. Mol. Sci..

[35] Ghasemitarei M., Privat-Maldonado A., Yusupov M., Rahnama S., Bogaerts A., Ejtehadi M.R. (2022). Effect of Cysteine Oxidation in SARS-CoV-2 Receptor-Binding Domain on Its Interaction with Two Cell Receptors: Insights from Atomistic Simulations. J. Chem. Inf. Model..

[36] Hoffmann M., Kleine-Weber H., Schroeder S., Krüger N., Herrler T., Erichsen S., Schiergens T.S., Herrler G., Wu N.-H., Nitsche A. (2020). SARS-CoV-2 Cell Entry Depends on ACE2 and TMPRSS2 and Is Blocked by a Clinically Proven Protease Inhibitor. Cell.

[37] Delcanale P., Uriati E., Mariangeli M., Mussini A., Moreno A., Lelli D., Cavanna L., Bianchini P., Diaspro A., Abbruzzetti S. (2022). The Interaction of Hypericin with SARS-CoV-2 Reveals a Multimodal Antiviral Activity. ACS Appl. Mater. Interfaces.

[38] Crocker L.B., Lee J.H., Mital S., Mills G.C., Schack S., Bistrović-Popov A., Franck C.O., Mela I., Kaminski C.F., Christie G. (2022). Tuning Riboflavin Derivatives for Photodynamic Inactivation of Pathogens. Sci. Rep..

[39] Enwemeka C.S., Bumah V.V., Castel J.C., Suess S.L. (2022). Pulsed Blue Light, Saliva and Curcumin Significantly Inactivate Human Coronavirus. J. Photochem. Photobiol. B.

[40] Pourhajibagher M., Azimi M., Haddadi-Asl V., Ahmadi H., Gholamzad M., Ghorbanpour S., Bahador A. (2021). Robust Antimicrobial Photodynamic Therapy with Curcumin-Poly (Lactic-Co-Glycolic Acid) Nanoparticles against COVID-19: A Preliminary in Vitro Study in Vero Cell Line as a Model. Photodiagn. Photodyn. Ther..

[41] Gendrot M., Andreani J., Duflot I., Boxberger M., Le Bideau M., Mosnier J., Jardot P., Fonta I., Rolland C., Bogreau H. (2020). Methylene Blue Inhibits Replication of SARS-CoV-2 in Vitro. Int. J. Antimicrob. Agents.

[42] Gomes M., Bartolomeu M., Vieira C., Gomes A.T.P.C., Faustino M.A.F., Neves M.G.P.M.S., Almeida A. (2022). Photoinactivation of Phage Phi6 as a SARS-CoV-2 Model in Wastewater: Evidence of Efficacy and Safety. Microorganisms.

[43] Sadraeian M., Junior F.F.P., Miranda M., Galinskas J., Fernandes R.S., da Cruz E.F., Fu L., Zhang L., Diaz R.S., Cabral-Miranda G. (2022). Study of Viral Photoinactivation by UV-C Light and Photosensitizer Using a Pseudotyped Model. Pharmaceutics.

[44] Duguay B.A., Herod A., Pringle E.S., Monro S.M.A., Hetu M., Cameron C.G., McFarland S.A., McCormick C. (2022). Photodynamic Inactivation of Human Coronaviruses. Viruses.

[45] Morimoto S., Rosin J.L.A., Matuck B.F., Schröter G., Rodrigues M.F.S.D., Ramalho K.M., Raggio D.P., Moreira M.S., da Silva L.F.F. (2022). APDT for Oral Decontamination of Hospitalized Patients with COVID 19. Photodiagn. Photodyn. Ther..

[46] Sachet P., Rocha B.A., Lima F.S., da Silva Pedrosa M., Guollo A., Melo Filho M.R., de Horta M.C.R., Simões A. (2022). Management of Orofacial Lesions with Antimicrobial Photodynamic Therapy and Photobiomodulation Protocols in Patients with COVID-19: A Multicenter Case Series. Photodiagn. Photodyn. Ther..

